# Three-Dimensional Genome Map of the Filamentous Fungus *Penicillium oxalicum*

**DOI:** 10.1128/spectrum.02121-21

**Published:** 2022-05-02

**Authors:** Cheng-Xi Li, Lin Liu, Ting Zhang, Xue-Mei Luo, Jia-Xun Feng, Shuai Zhao

**Affiliations:** a State Key Laboratory for Conservation and Utilization of Subtropical Agro-bioresources, Guangxi Research Center for Microbial and Enzyme Engineering Technology, College of Life Science and Technology, Guangxi University, Nanning, Guangxi, China; b Anhui Key Laboratory of Infection and Immunity, Department of Microbiology and Parasitology, Bengbu Medical College, Bengbu, Anhui, China; c Wuhan Frasergen Bioinformatics Co., Ltd., Wuhan, Hubei, China; Jawaharlal Nehru Centre for Advanced Scientific Research

**Keywords:** three-dimensional genome, *Penicillium oxalicum*, chromatin interaction, globule, cellulase

## Abstract

Higher-order spatial organization of the chromatin in the nucleus plays crucial roles in the maintenance of cell functions and the regulation of gene expression. Three-dimensional (3D) genome sequencing has been used to great effect in mammal and plants, but the availability of 3D genomes of filamentous fungi is severely limited. Here, we performed a chromosome-level genome assembly of Penicillium oxalicum through single-molecule real-time sequencing (Pacific Biosciences) and chromatin interaction mapping (Hi-C), with a scaffold *N*_50_ of 4.07 Mb and a contig *N*_50_ of 3.81 Mb, and further elucidated the 3D genome architecture of P. oxalicum. High-frequency interchromosomal contacts occurred within the centromeres and telomeres, as well as within individual chromosomes. There were 12,203 *cis*-interactions and 7,884 *trans*-interactions detected at a resolution of 1 kb. Moreover, a total of 1,099 topologically associated domains (or globules) were found, ranging in size from 2.0 to 76.0 kb. Interestingly, transcription factor-bound motifs were enriched in the globule boundaries. All the cellulase and xylanase genes were discretely distributed in the 3D model of the P. oxalicum genome as a result of few *cis-* and *trans*-interactions. Our results from this study provide a global view of chromatin interactions in the P. oxalicum genome and will act as a resource for studying spatial regulation of gene expression in filamentous fungi.

**IMPORTANCE** The spatial structure of chromatin plays important roles in normal cell functions and the regulation of gene expression. The three-dimensional (3D) architectures of the genomes of many mammals and plants have been elucidated, but corresponding studies on filamentous fungi, which play vital roles as decomposers of organic matter in the soil, are very limited. Penicillium oxalicum is one of the predominant cellulolytic aerobic fungi in subtropical and tropical forest soils and can secrete integrative cellulase and xylanase under integrated regulatory control, degrading plant biomass highly efficiently. In the present study, we employed Hi-C technology to construct the 3D genome model of P. oxalicum strain HP7-1 and to further investigate cellulase and xylanase as well as transcription factor genes in 3D genome. These results provide a resource to achieve a deeper understanding of cell function and the regulation of gene expression in filamentous fungi.

## INTRODUCTION

Eukaryotic chromosomes are highly ordered and compactly packaged within the nucleus, an organization that is associated with important nuclear processes, including DNA replication and repair, gene transcription, and regulation of gene expression (1). Chromosome conformation capture (3C) combined with next-generation DNA sequencing (Hi-C) has been developed and widely used for establishment of three-dimensional (3D) genome sequences, revealing the true spatial structure and interactions, dynamic changes, and biological functions of chromatin (2). Studies of the 3D genome provide novel insights into cell biology, molecular biology, and genetics.

Eukaryotic genomic DNA is hierarchically folded into chromosome territories or subdomains, topologically associated domains (TADs), and chromatin loops at multiple scales. Among them, TADs are identified as functional genome-organizing units on a submegabase scale related to important structural and regulatory roles, which operate through an active and well controlled process rather than occurring randomly (3, 4). Both condensin and cohesin have been found to be implicated in TAD organization, although the mechanism has not been clarified yet (4). The role of TADs is well known in some eukaryotes, such as humans and Drosophila, but is less well understood in yeast and plants, which may be due to the lack of homologs of mammalian insulator proteins, such as the CCCTC-binding factor (1, 5). On the other hand, genome organization in the yeast Schizosaccharomyces pombe and Arabidopsis thaliana is associated with heterochromatic interaction (6, 7).

Although there is considerable literature on the 3D genome architecture in mammals and plants (1, 7, 8), less is known in filamentous fungi. In the sole report to date, the 3D organization of the Neurospora crassa genome is dominated by heterochromatic interactions, known as “heterochromatin bundling.” Blocks of heterochromatin divide the genome into numbers of TADs, also called “self-associated chromosomal domains” or “globules” (9). Fungal chromosomes and chromatin are highly diverse, even within a single species (10). Therefore, it will be of great value to understand the 3D genomic organization of other filamentous fungi.

Penicillium is widely distributed in various natural habitats, including terrestrial and marine environments (11–14), consisting of 483 accepted species (15). Among them, Penicillium oxalicum can secrete large amounts of carbohydrate-active enzymes (CAZymes) to digest plant biomass and produce secondary metabolites with a variety of biological activities, including antimicrobial and antitumor compounds (12), which have considerable potential for use in the biorefinery, bioremediation, and pharmaceutical industries.

The National Center for Biotechnology Information (NCBI) revealed that fewer than one-tenth of Penicillium strains have had their genome sequenced, with most of the sequenced genomes being at the draft level. Among the Penicillium strains that have been sequenced, six P. oxalicum strains (114-2, HP7-1, SYJ-1, JU-A10-T, TY02, and SGAir0226) are available (16–19), of which strain JU-A10-T was derived from wild-type 114-2 (20). However, the sequences obtained are insufficient to reach the requirements for analysis of the 3D chromatin architecture.

P. oxalicum strain HP7-1, isolated from a subtropical forest soil in China, generates high cellulase activity against pretreated sugarcane bagasse (17), a process that has industrial potential. In the present study, we reported a high-quality chromosome-level genome sequence of P. oxalicum HP7-1, using a combination of Pacific Biosciences (PacBio) sequencing and Hi-C chromosome conformation capture sequencing technology. Furthermore, the 3D structure of strain HP7-1 chromosomes was revealed, and the sequence interactions of cellulase and xylanase genes, as well as genes encoding putative transcription factors, were investigated.

## RESULTS AND DISCUSSION

### Chromosome-level genome assembly of P. oxalicum.

The genome sequence of P. oxalicum HP7-1 had previously been obtained using an Illumina HiSeq 2000 system (accession number JRVD00000000 in GenBank) with a scaffold *N*_50_ of 3.3 Mb and a contig *N*_50_ of 0.5 Mb, which is comparable to that of the genome sequence of other P. oxalicum strains, such as 114-2 (16) uploaded on NCBI. However, the quality of the previously reported P. oxalicum genome sequences was insufficient to be analyzed by Hi-C technology. In the current study, the genome of strain HP7-1 was further upgraded using the PacBio sequencing platform, with the assistance of the Hi-C technique for scaffolding. A total of 10.14 Gb clean subreads with an *N*_50_ size of 11.37 kb and average length of 9,888 bp was generated. The subread lengths ranged from 1.0 to 146.72 kb. After optimization of subread assembly by Falcon (version 0.3.0; https://github.com/PacificBiosciences/falcon) and Celera assembler (version 8.3; http://sourceforge.net/projects/wgs-assembler/files/wgs-assembler/wgs-8.3/), a 30.79-Mb genome of P. oxalicum, in accordance with previously reported genome sizes for this species (16–19), was generated, with 329-fold coverage. This initial genome was composed of 23 contigs with an *N*_50_ value of 3.81 Mb, with various lengths ranging from 4.18 to 5.87 Mb.

**FIG 1:**
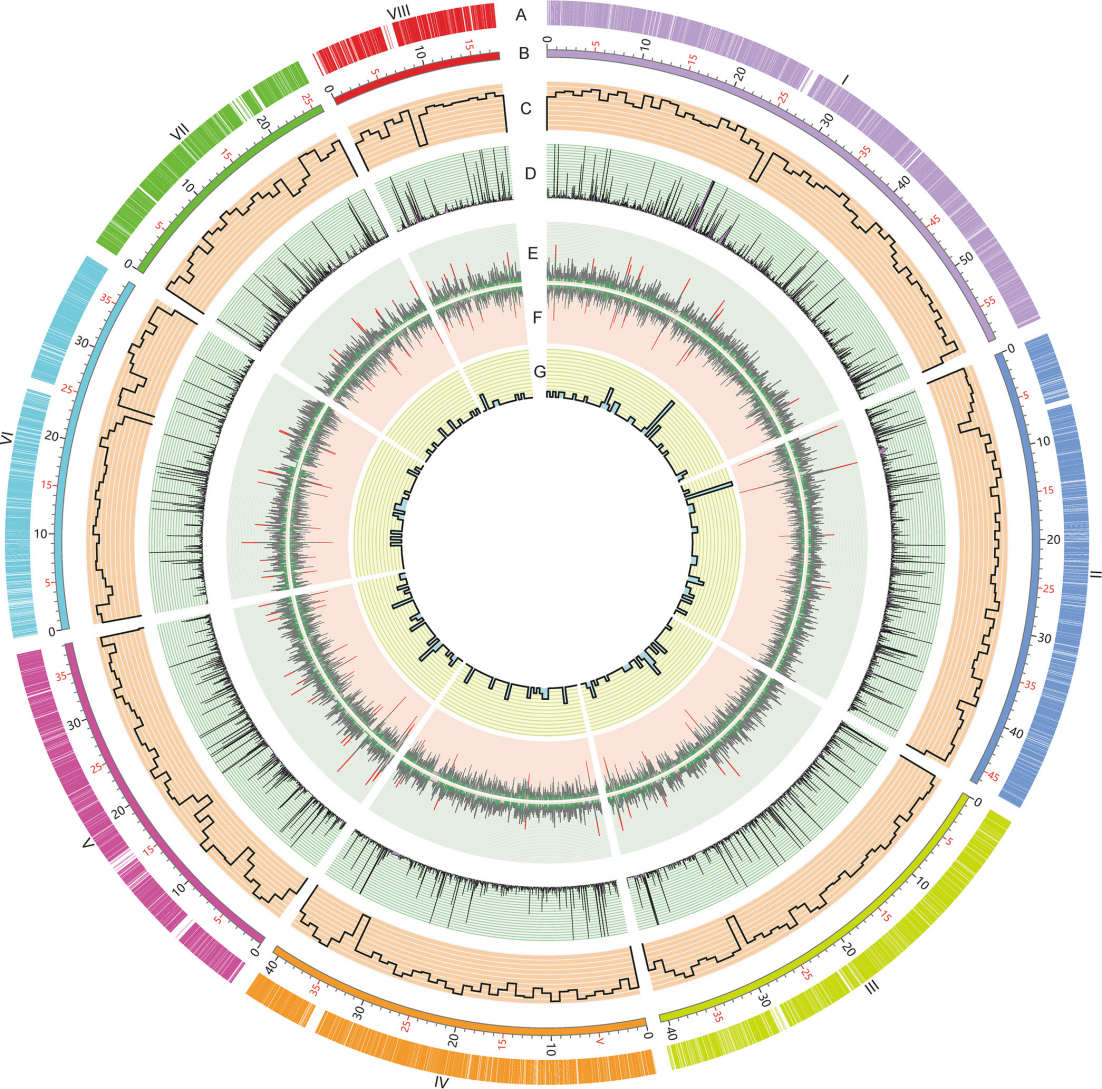
Circular map indicating the genomic features of P. oxalicum genome. (A) Locations of predicted genes. The Roman numbers represent the chromosomes. (B) Schematic representation of chromosomal size (Mb scale). (C) Gene density represented by number of genes in 100-kb nonoverlapping windows. (D) Gene expression under Avicel induction. (E) Exon numbers in protein-coding genes. Red lines represent exon number (>10) in a gene, whereas green color indicates one exon contained. (F) Intron numbers in protein-coding genes. Red lines represent intron number (>10) in a gene, whereas green indicates no intron contained. (G) Number of tRNAs in 100-kb nonoverlapping windows.

Hi-C technology was used to correct instances of misjoining and to reorder and reorientate the contig assembly. An average of 116 million valid reads was produced per replicate for use in further Hi-C analysis, accounting for 55.97% of the total clean reads mapped onto the genome of P. oxalicum. Analysis by Genome DISCO (20) revealed a correction coefficient of 0.976 between two replicates, thus suggesting that the Hi-C data were reproducible and hence suitable for further analyses. Finally, eight pseudochromosomes with lengths of 1.80 to 5.87 Mb were generated, assembled by using 13 contigs (Fig. 1A). The telomeric repeats (5′-TTAGGGG-3′)*_n_* were detected at the ends of all pseudochromosomes except for the 3′-end of chromosome VIII. The scaffold *N*_50_ reached 4.07 Mb, with a contig *N*_50_ of 3.81 Mb. The total length of all pseudochromosomes was 30.64 Mb, accounting for 99.51% of the estimated P. oxalicum genome. In addition, the average GC content of the P. oxalicum genome was 50.69% (Table 1). The Benchmarking Universal Single-Copy Orthologs (BUSCO) assay revealed a total of 286 (98.60%) complete gene models among 290 conserved genes in the fungi_odb9 data set (https://busco-archive.ezlab.org/v2/), suggesting a high level of contiguity and sequence quality of the final genome assembly.

**TABLE 1:** Chromosome-level genome features of P. oxalicum strain HP7-1^*a*^

Genome features	Value
Chromosome features
No. of scaffolds	8
No. of contigs	13
Size of scaffolds (Mb)	30.64
GC content of scaffolds (%)	50.69
*N*_50_ scaffold length (bp)	4,068,681
*N*_90_ scaffold length (bp)	2,593,408
Maximum scaffold length (bp)	5,870,233
Minimum scaffold length (bp)	1,795,220
Avg no. of contigs per scaffold	1.63
Protein-coding genes of scaffolds	9,728
Avg gene length (bp)	1,615.07
Avg CDS length (bp)	1,412.63
GC content of CDS sequences (%)	54.44
Genome features	
Avg no. of exons per gene	2.94
Avg no. of introns per gene	1.94
Complete BUSCOs of genome (%)	98.6
rRNAs	45
tRNAs	201

*^a^*BUSCO, Benchmarking Universal Single-Copy Orthologs; CDS, coding DNA sequence.

### Genome annotation.

A combination of *de novo* and homology predictions was used for annotation of the P. oxalicum genome. A total of 9,728 protein-coding genes was predicted (Table S1). Of these genes, 5,359 (55.08%), 7,141 (73.40%), 3,148 (32.36%), 2,061 (21.19%), and 4,018 (41.30%) were annotated using the Gene Ontology (GO), InterPro, UniProt, euKaryotic Orthologous Groups of proteins, and Kyoto Encyclopedia of Genes and Genomes (KEGG) databases, respectively. Relative to the proteins annotated by the Illumina HiSeq 2000 system (17), 85 novel hypothetical proteins were found. Notably, of the 9,728 proteins, 712 CAZymes, 496 putative transcription factors, and 47 clusters associated with secondary metabolism were included (Table S1), which are comparable with the previously reported (17, 21). Furthermore, 45 rRNAs (38 5S rRNAs, 2 5.8S rRNAs, 3 18S rRNAs, and 2 28S rRNAs) and 201 tRNAs were identified via rRNAmmer (version 1.2) and tRNAscan-SE (version 2.0.9), respectively (22, 23). The general features of P. oxalicum strain HP7-1 genome are presented in Table 1.

### 3D genome organization of P. oxalicum.

**FIG 2:**
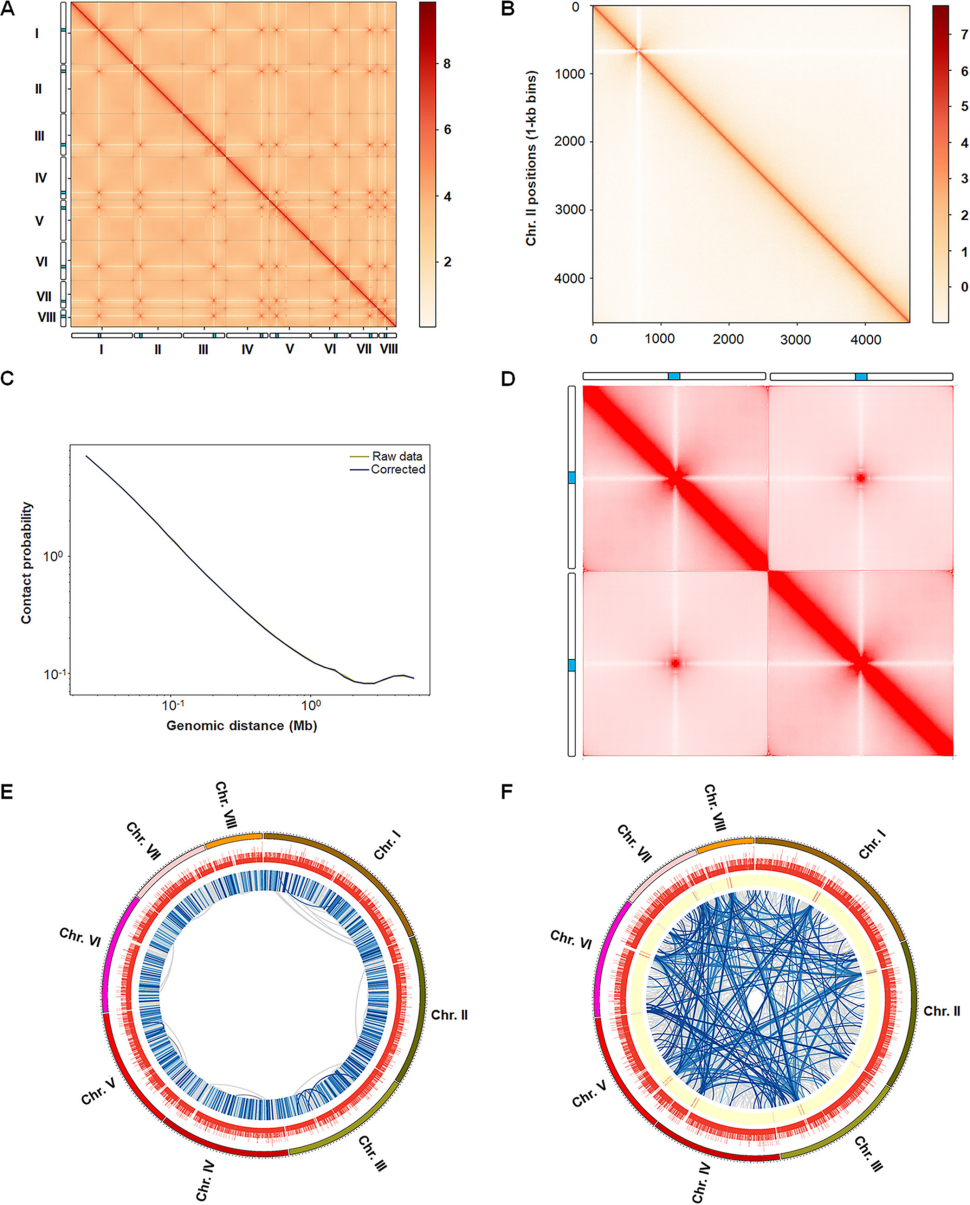
Hi-C contact map and genome-wide interaction matrix of P. oxalicum. (A) Hi-C interactome (10-kb bins) within and among chromosomes (I to VIII). The color intensity represents the contact frequency. The columns beside the *x* and *y* axes indicate chromosomes, in which the blue color represents the predicted centromeres. (B) Heat map showing Hi-C interaction of chromosome II (1-kb min). (C) Genome-wide contact decay curve. (D) Aggregate chromosome analysis on Hi-C map of P. oxalicum, which helps highlight chromosome-scale features associated with a collection of chromosomes. This plot symmetrizes across all possible orders and orientations of individual chromosomes to generate representative isochromosome-by-isochromosome map showing the strong Rabl-like features. The columns beside the plot indicate virtual chromosomes, in which the blue color represents the centromeres. (E, F) Circos diagrams indicating the genome-wide significant *cis*- and *trans*-interactions, respectively. In panel E, the tracks (from outside to inside) indicate chromosome, gene number, and interaction links, the color of which becomes darker as the *P* value increases. In panel F, the tracks (from outside to inside) indicate chromosome, gene number, and enrichment degree of number of *trans*-interactions, where the red color indicates *trans*-interactions, and the interaction links, in which the color becomes darker as the number of reads increases.

To understand the 3D genome architecture of P. oxalicum, Hi-C data were further analyzed. We generated genome-scale Hi-C interaction heat maps at resolutions of 20, 10, 2, and 1. The heat maps revealed much more frequent intrachromosomal interactions than interchromosomal interactions (Fig. 2A and B). Contact decay curves revealed fewer long-range interactions than short-range contacts within chromosomes (Fig. 2C). It was noted that high-frequency interchromosomal contacts occurred within the pericentromeric and subtelomeric regions, including centromeres and telomeres, of all eight chromosomes, as well as in intrachromosomal contacts (Fig. 2A and D), findings that are comparable with those from N. crassa (9). To further determine the chromosome-scale folding features, aggregate chromosome analysis indicated that the 3D genome architecture of P. oxalicum belonged to type I with the Rabl-like feature of centromere clustering, as described by Hoencamp et al. (24), clearly exhibiting enhanced contact frequency between centromeres and telomeres (Fig. 2D).

**FIG 3:**
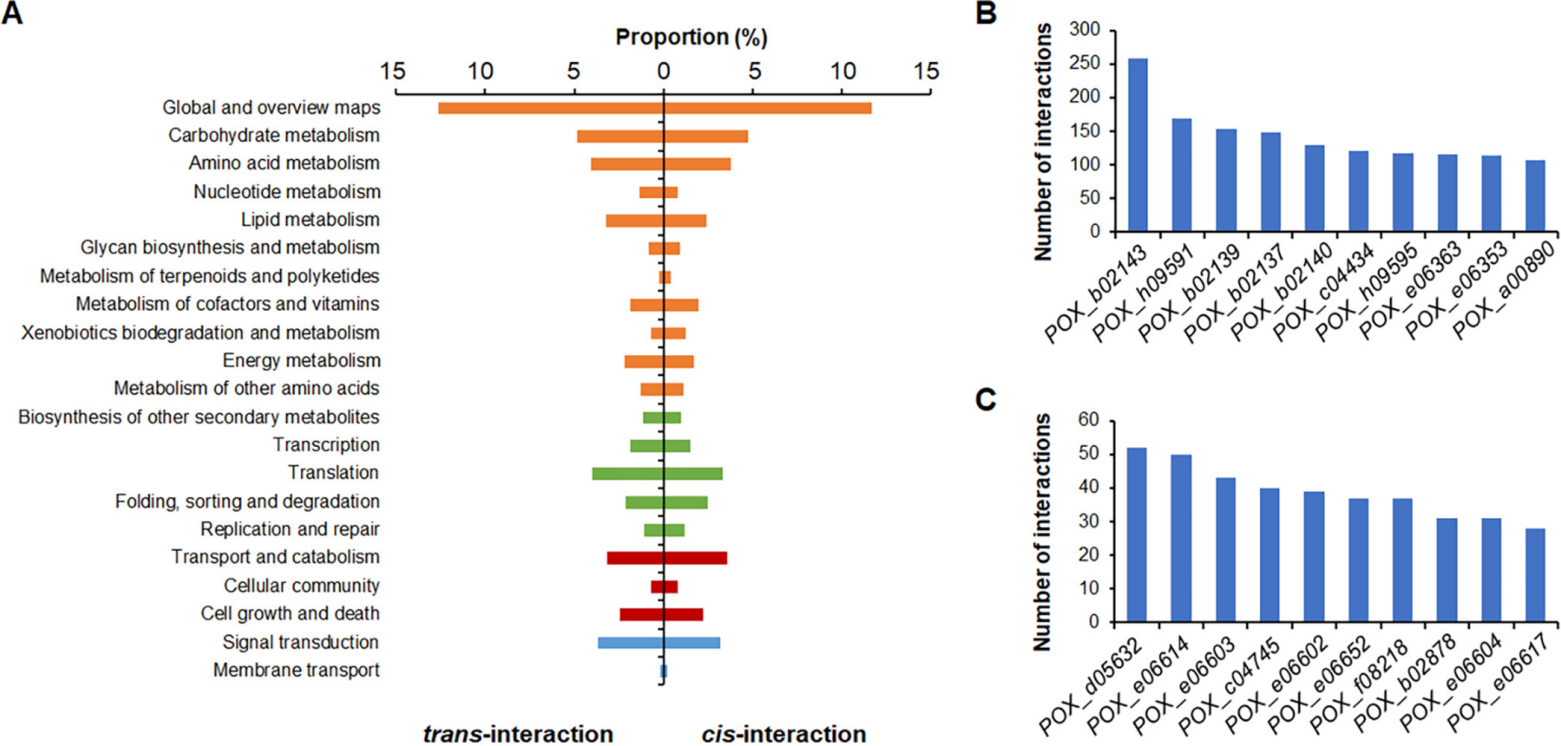
Kyoto Encyclopedia of Genes and Genomes analysis of genes with *cis*- and *trans*-interactions (A) and the top 10 genes with the greatest number of *cis*- (B) and *trans*-interactions (C). In panel A, the orange bar charts indicate genes involved in metabolism; the green bar charts are genes participating in genetic information processing; the dark red bar charts are genes involved in environmental information processing; and the blue bar charts represent genes involved in cellular processes.

At a resolution of 1-kb, Fit-Hi-C analysis (25), a tool for assigning statistical confidence estimates to chromosomal contact heat maps, detected 12,203 *cis*-interactions (Fig. 2E; Table S2) and 7,884 *trans*-interactions (Fig. 2F; Table S3), with thresholds of *q* value ≤ 0.01 and number of interacted reads > 2. The frequency of *cis*-interaction was negatively correlated with DNA distance (Fig. S1). Furthermore, DNA fragments with *cis*-interactions and *trans*-interactions covered 5,738 and 1,324 genes, respectively. Of these genes, 967 genes exhibited both *cis*- and *trans*-interactions. KEGG annotation indicated that 30.7% of the 5,738 genes with *cis*-interactions were involved in metabolism, followed by genetic information processing (8.5%) and cellular processes (6.6%), whereas 33.3% of the 1,322 genes with *trans*-interactions were involved in metabolism. Comparative analysis revealed that the genes involved in nucleotide and energy metabolisms that exhibited *cis-*interactions were significantly fewer than those exhibiting *trans*-interactions, whereas the genes participating in xenobiotic biodegradation and metabolism that exhibited *cis-*interactions were more frequent than those with *trans*-interactions (Fig. 3A). Of these genes, the three most highly connected (Hub) genes with *cis*-interactions were *POX_d05632* (52 interactions), *POX_e06614* (50 interactions), and *POX_e06603* (43 interactions), whereas the three most highly connected genes with *trans*-interactions were *POX_b02143* (258 interactions), *POX_h09591* (169 interactions), and *POX_b02139* (154 interactions) (Fig. 3B and C). Of these genes, *POX_d05632* and *POX_e06614* encoded AMP-dependent synthetase/ligase and vacuolar fusion protein CCZ1, respectively, whereas *POX_e06603* encoded a basic leucine zipper (bZIP) transcription factor. *POX_b02143* and *POX_h09591* encoded the ABC multidrug transporter AtrF and the AP-3 complex subunit involved in sorting of PQ-loop-family amino acid transporters to the vacuolar/lysosomal membrane, respectively (26), whereas POX_b02139 was annotated as a hypothetical protein (Table S1).

**FIG 4:**
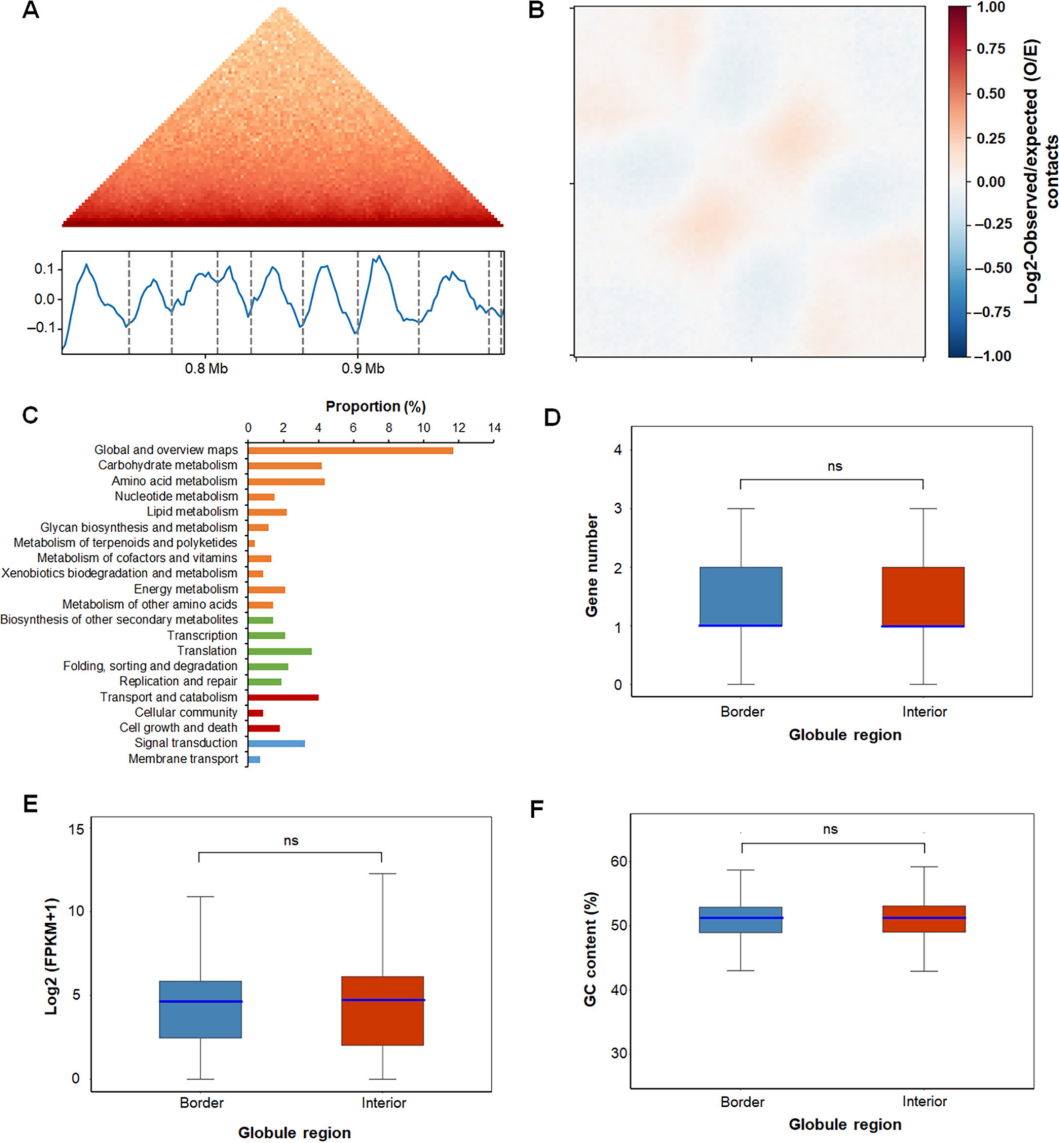
Globule analysis in 3D architecture of P. oxalicum. (A) Hi-C interaction matrix of a region (Chr. III 704000 to 992000) indicating globules. (Top) Hi-C interaction matrix; (bottom) globule boundaries (vertical bars) and insulation scores. The vertical axis is the insulation score, whereas the gray dashed line represents the globule boundary. (B) Globule structure polymeric analysis. (C) Kyoto Encyclopedia of Genes and Genomes analysis of genes localized in globule boundaries. (D) Distribution of gene number in each bin included in globule interiors and borders. (E) Gene expression in globule interiors and borders through transcription analysis. (F) GC content of globule interiors and borders. In panels D to F, the light blue line represents the median. “ns” represents no significant difference between globule interior and border, analyzed by the Wilcoxon test.

The TAD is considered to be the basic 3D genome-organizing unit in mammals and plants and is also called a “globule” in fungi, such as N. crassa and S. pombe. Commonly, TADs are defined as self-associating chromosomal domains at the submegabase scale, whereas globules are defined as locally self-interacting domains of 50 to 100 kb in size (9, 27, 28). Screening each chromosome at a resolution of 2 kb using insulation score (29) revealed 1,099 globules in the P. oxalicum 3D genome, ranging in size from 2.0 to 76.0 kb (Table S4; Fig. 4A). Polymeric analysis of globule structure (30) further showed that the self-interactions were separated by insulating boundaries (Fig. 4B). There were 1,282 and 9,476 genes localized in the boundaries and interiors of the globules, respectively, of which 1,051 genes were found across both the boundaries and the interiors. KEGG annotation indicated that 32.6% of these 1,051 genes were involved in metabolism, followed by genetic information processing (9.9%) and cellular processes (6.7%) (Fig. 4C). Neither the density (Fig. 4D) nor the transcription (Fig. 4E) of genes in each bin, including in globule boundary and interior regions or GC contents (Fig. 4F) was detected.

**FIG 5:**
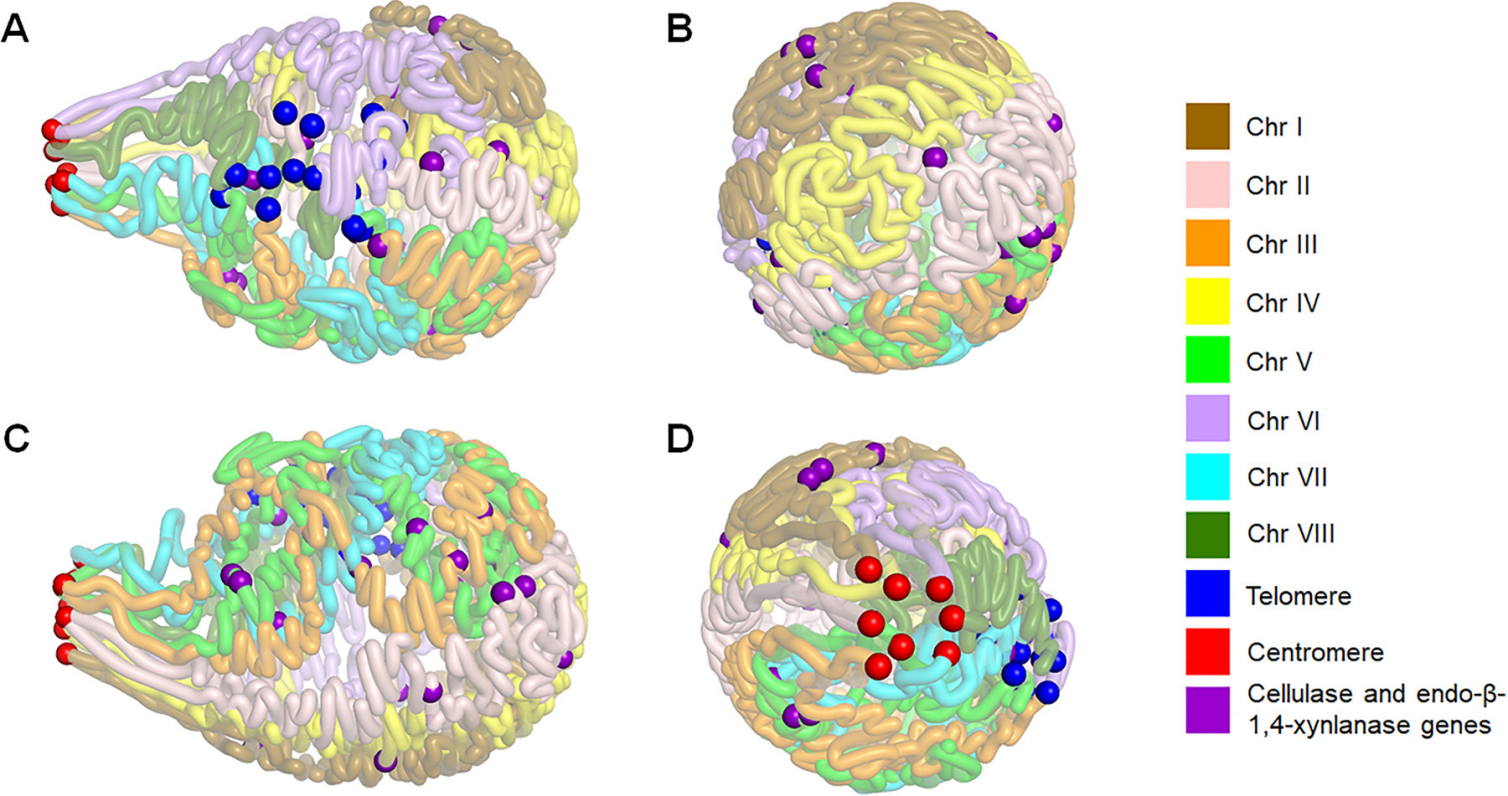
3D model of P. oxalicum genome under Avicel induction. (A) Front view. (B) Right flank view. (C) Back view. (D) Left flank view. Red, blue, and purple balls represent the predicted centrosomes, telomeres, and cellulase and endo-β-1,4-xylanase genes, respectively.

Based on the data above, we carried out *in silico* modeling of a 3D map of the P. oxalicum genome. The resulting map resembled a light bulb, comprising clustered centromeres as the “socket base” and 16 chromosome arms forming the “bulb.” All the telomeres were grouped together, localized on the side of the bulb. Each chromosome conformation supported a clothes-pin like structure, which was also observed in N. crassa chromosomes (9), with the centromere at one end of the folded chromosome and the telomeres almost at the opposite end (Fig. 5).

### 3D organization of cellulolytic and xylanolytic enzyme genes in the P. oxalicum genome.

**FIG 6:**
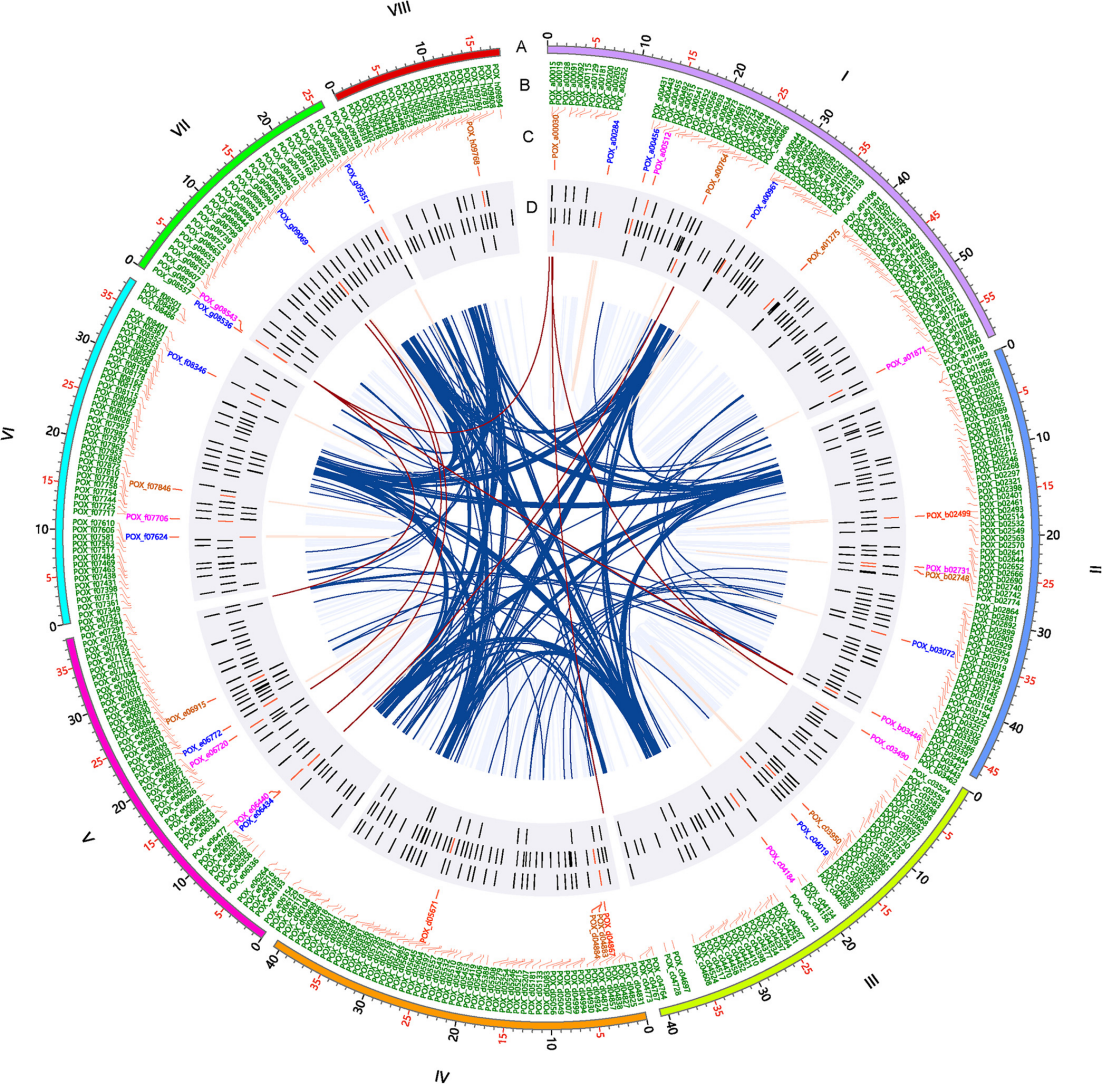
Circos plot indicating interactions of cellulase and xylanase genes and transcription factor genes in P. oxalicum. (A) Schematic representation of chromosomal size (Mb scale). (B) Names of transcription factor genes. (C) Names of genes encoding cellulase and xylanase. (D) Location of the genes described above. The roman numbers represent the chromosomes. Red gene ID, cellobiohydrolase gene; brown gene ID, endo-β-1,4-glucanase; blue gene ID, β-glucosidase; pink gene ID, endo-β-1,4-xylanase. The brown and dark blue lines represent contacts among enzyme and transcription factor genes.

In the genome of P. oxalicum obtained, 25 cellulase (three cellobiohydrolases [CBHs], 10 endo-β-1,4-glucanases [EGs], and 12 β-glucosidases [BGLs]) and 10 endo-β-1,4-xylanase (XYNs) genes were annotated, a result that was comparable to that reported from the previously reported P. oxalicum genome (17). These genes were nonuniformly distributed across the eight chromosomes. Eight of the genes were localized on chromosome I, followed by five each on chromosomes II and V. The major cellobiohydrolase genes *cbh1* and *cbh2* were located on chromosome IV (Fig. 6). These cellulase and XYN genes were distributed into distinct globules, except for *POX_g08536* and *POX_g08543* in Globule 938 and *POX_d4883* and *POX_d4884* in Globule 516. Three genes, *POX_a00284*, *POX_b03072*, and *POX_f07624*, spanned globule boundaries and interiors (Table S1).

In addition, 15 genes, consisting of seven *bgl*, one *cbh*, five *eg*, and two *xyns*, exhibited *cis*- interactions. Six genes (*eg* gene *Cel5C*/*POX_a00030*; *bgl* genes *POX_e06434*, *POX_g08536* and *POX_g09069*; and *xyn* genes *xyn11A*/*POX_c03490* and *POX_c03490*) were found to exhibit both *cis*- and *trans*-interactions. *cis*-Interacted DNA fragments were localized in adjacent up- and/or down-flanking regions of each gene, including promoter and terminator regions. For example, the *bgl* gene *POX_g09351* could make contact with DNA fragments associated with its predicted promoter and terminator regions, *POX_g09352* and *POX_g09357*, respectively. With respect to the *trans*-interactions, the *bgl* gene *POX_g08536* made contact with DNA fragments associated with *POX_b03474*, *POX_b03469*, and *POX_e07327*, and Chr I34,000 to 35,000. Of these, *POX_b03469* and *POX_e07327* were annotated as AMP-dependent synthetase/ligase and sulfite oxidase, respectively, whereas *POX_b03474* was a hypothetical protein. In contrast, each of the other genes made contact with only one 1-kb DNA fragment. For example, the *xyn* gene *POX_e06720*/*xyn11A* interacted with a DNA fragment referred to as *POX_g09033*, encoding the translation initiation factor SUI1. In contrast, the key cellulase genes, such as *POX_d04867*/*cbh1*, *POX_d05671*/*cbh2*, and *POX_d04883*/*eg1*, did not form interactions. No interactions were found between cellulase genes and xylanase genes (Fig. 6; Tables S2 and S3). Those *trans*-interactions possibly resulted from overall genomic proximity of centromeres and telomeres, but it could not be excluded that they may be due to the induction in response to specific carbon source Avicel or coaffection by the proximity and induction. When cultivated in Avicel medium, major cellulase and xylanase genes in P. oxalicum were coexpressed (17, 31), but with a low-frequency DNA interaction, causing speculation that the biosynthesis of cellulases and xylanases was conditionally and specifically controlled, which might have happened during the transcription process resulted by a series of factors such as transcription factors (TFs).

### 3D organization of putative TF-encoding genes in P. oxalicum.

It is important to know how TF-encoding genes are spatially organized in P. oxalicum. TFs play essential roles in promoting the expression of cellulase and xylanase genes (32). We hypothesized that spatial interactions affected the expression of some TF-encoding genes. In the assembled genome, 496 TFs were predicted to be distributed into 430 globules. Among them, (partial) DNA sequences of 77 TF genes were localized at globule boundaries. There were 346 and 79 TF genes with *cis*- and *trans*-interactions, respectively, 64 of which shared both types of interaction (Tables S2 and S3).

The regulation of expression of the genes encoding the key TFs known to regulate the expression of cellulase and xylanase genes in P. oxalicum needs to be investigated for this biomass-degrading fungus, such as *clrB/POX_b01969*, *cxrA/POX_a00763*, *cxrB/POX_f07563*, *atf1/POX_b03027*, *amyR/POX_f08097*, *xlnR/POX_d05133*, *cbh/POX_e06803*, *creA/POX_e07192*, *nsdD/POX_g08615*, and *mbf1/POX_g08739*. Expression of these genes showed changes to various degrees at the transcriptional level in response to nonpreferred carbon sources, including Avicel, in comparison with that on glucose (31, 33–36). All the investigated genes, except *atf1/POX_b03027* and *amyR/POX_f08097*, exhibited found *cis*-interactions with adjacent up- and/or down-flanking regions of each gene. In contrast, only two genes *trans*-interacted with DNA fragments, namely, *cxrA/POX_a00763* with *POX_c04329* and *amyR/POX_f08097* with *POX_b02143*, *POX_b02170*, *POX_e06384*, *POX_g09184*, and *POX_g09188*, respectively (Fig. 6). Protein annotation revealed that POX_b02143, POX_g09184, and POX_g09188 are ABC multidrug transporter AtrF, glutathione transporter 1, and fatty acid synthase subunit alpha, respectively, whereas the others are hypothetical proteins (Table S1). Whether these interactions affect the expression of the TF-encoding genes described above needs to be further determined. In addition, it should be noted that the interactions involving TF-encoding genes were infrequent, comparable to the interactions involving cellulase and xylanase genes.

### TF-binding motifs are enriched at the globule boundaries.

TAD/globule borders are generally rich in TF-binding specific motifs and heterochromatic marker-enriched regions, such as H3K9me3 and H3K27me2/3 (37). In mammalian genomes, the CCCTC-binding factor CTCF is responsible for TAD organization and is frequently colocalized in “convergent” CTCF sites with cohesin, one of the components of structural maintenance of chromosomes (SMC) complexes (38). However, the interaction of TFs, such as ACE2 and Ams2, with SMC complexes may predominantly contribute to higher-order genome architecture in yeasts because CTCF is absent (4, 39).

In the current study, the MEME Suite (http://meme-suite.org) was employed to screen motifs in the globule boundaries of P. oxalicum strain HP7-1, using the TF-binding profile of fungi in the JASPAR database (http://jaspar.genereg.net). A total of 163 candidate motifs were detected at globule boundaries, representing the potential binding sites of 163 TFs from S. cerevisiae and N. crassa. Unexpectedly, homologous alignment studies indicated the presence in P. oxalicum of 93 orthologs, with identities of 22% to 90% (Table S5).

**FIG 7:**
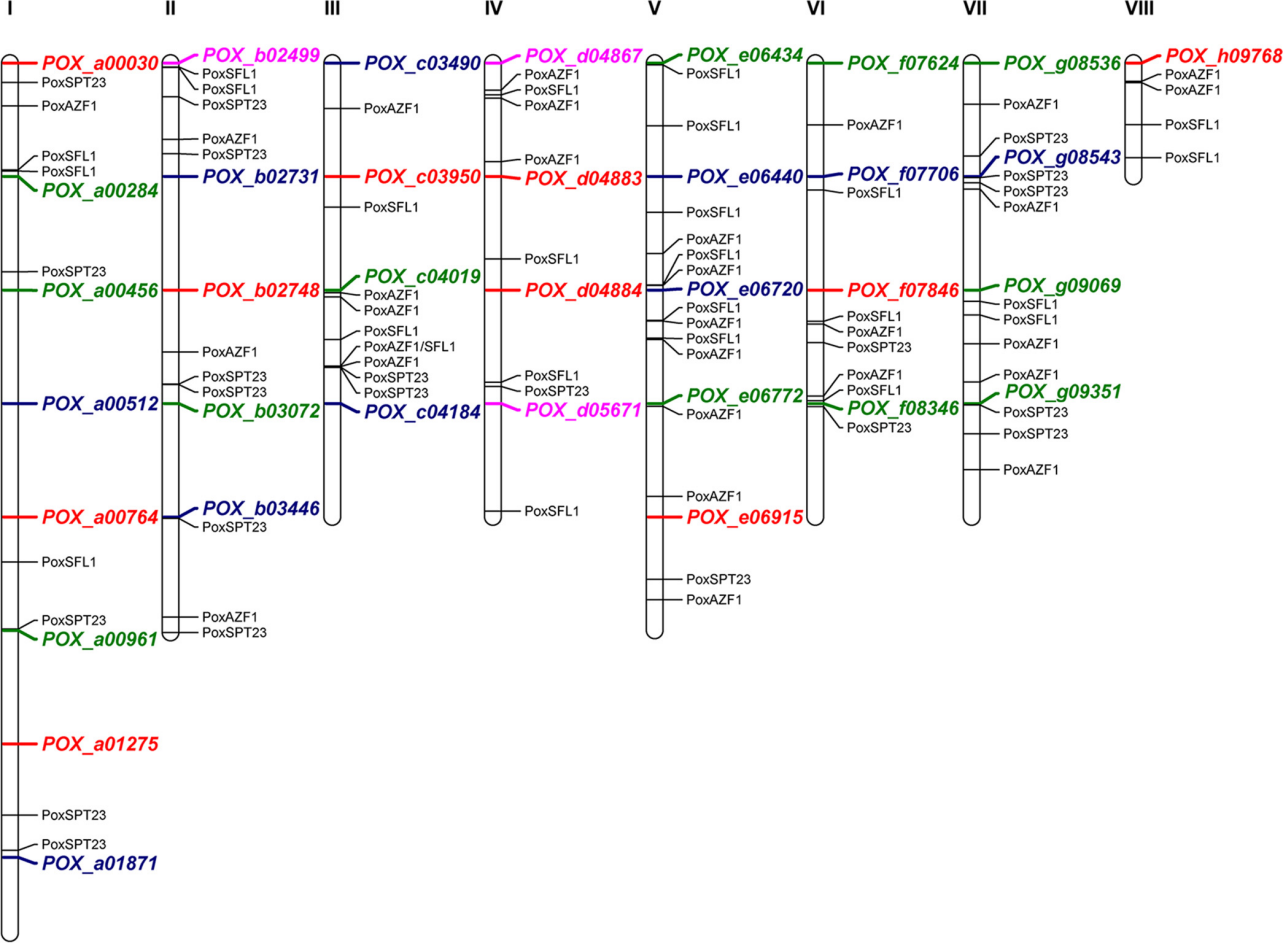
Motif enrichment analysis of three transcription factors, PoxAZF1, PoxSFL1, and PoxSPT23, in the promoter regions of cellulase and xylanase genes in P. oxalicum. These transcription factor-binding DNA motifs were found in the globule boundaries, and the numbers of motifs bound by them were ranked as the top three among all the identified transcription factors. Gene IDs in pink, red, green, and blue color represent cellobiohydrolase genes, endo-β-1,4-glucanase, β-glucosidase, and endo-β-1,4-xylanase genes, respectively. The roman numbers represent the chromosome number. The interval between each successive two genes indicates the 1.5-kb upstream sequence before the start codon (ATG) of the upper gene.

Of these proteins, POX_c04470, POX_c04124, and POX_b02187 ranked as the top three proteins in the “hierarchy” of the proportion of the TF motif-containing globule boundary number to the total globule boundary number, with values of 65.99, 53.25, and 50.69%, respectively. POX_c04470, POX_c04124, and POX_b02187 were identified as the homologous proteins of AZF1, SPT23, and SFL1 through NCBI BLASTP, with identities of 48.5, 28.9, and 45.4%, respectively, which were thereby redesignated PoxAZF1, PoxSPT23, and PoxSFL1. The function of AZF1, an asparagine-rich Zn2Cys6 protein, is dependent on carbon sources; AZF1 is required for the growth and division of S. cerevisiae in the presence of glucose, although it switches to the maintenance of cell wall integrity in the presence of glycerol-lactate (40). Recently, TrAZF1 has been shown to regulate the expression of major cellulase genes such as *cbh1* and *cel45a* in the filamentous fungus Trichoderma reesei by directly binding to their promoters (41). MEME Suite screening detected the PoxAZF1-binding motif in the promoter regions (1.5 kb upstream) of several cellulase genes, including two *cbh* genes, *POX_b02499* and *cbh1*/*POX_d04867*; four *eg* genes, *Cel5C*/*POX_a00030*, *POX_e06915*, *POX_f07846*, and *Cel12A*/*POX_h09768*; seven *bgl* genes, *POX_b03072*, *POX_c04019*, *bgl1*/*POX_e06772*, *POX_f07624*, *POX_g08536*, *POX_g09069*, and *POX_g09351*; and five *xyn* genes, *POX_b02731*, *POX_c03490*, *POX_e06440*, *xyn11A*/*POX_e06720*, and *xyn11B*/*POX_g08543* (Fig. 7). These findings suggested that PoxAzf1 played an important role in the expression of cellulase and xylanase genes in P. oxalicum, as well as TrAZF1 in *T. reesei* (41).

Interestingly, MEME Suite analysis showed that another two TFs, PoxSPT23 and PoxSFL1, were able to bind to the promoter regions of major cellulase and xylanase genes, such as *cbh1* and *eg1* (Fig. 7). SPT23 regulates lipid desaturation and ergosterol biosynthesis, playing an important role in the ability of yeast cells to adapt to environmental changes (42, 43). SFL1 is involved in acidic biofilm formation and hyphal development in the pathogenic yeast Candida albicans (44, 45) and in determining virulence and heat tolerance in the phytopathogenic filamentous fungus Magnaporthe oryzae (46). To date, there have been no reports of the effects of SPT23 and SFL1 on the expression of cellulase and xylanase genes in fungi, and further study is therefore needed.

### Conclusions.

This study has provided the first opportunity to present a chromosomal-level genome of P. oxalicum and its 3D architecture. High-frequency interactions happened between the centromeres and the telomeres. There were 1,099 globules detected in the 3D genome sequence. Enriched at the globule boundaries were many motifs that bound TFs, such as PoxAZF1, PoxSPT23, and PoxSFL1. In addition, all the cellulase and xylanase genes were discretely distributed across the 3D genome model of P. oxalicum, with few *cis-* and *trans*-interactions, as well as the known TFs regulating the expression of cellulase and xylanase genes. This study has provided a global view of fungal chromatin interactions and a resource for studying spatial regulation of gene expression in filamentous fungi.

## MATERIALS AND METHODS

### Fungal materials and DNA extraction.

P. oxalicum strain HP7-1 was deposited in the China General Microbiological Culture Collection with the accession number 10781 and cultured on potato-dextrose-agar (PDA) at 4°C for temporary preservation. Asexual spores were collected using Tween 80 from a PDA plate inoculated with strain HP7-1 and cultured for 6 days at 28°C; spores were stored at –80°C in 50% (vol/vol) glycerol for long-term storage. To obtain the hyphae, fresh spores of strain HP7-1 were inoculated into glucose medium (1.0 g/liter glucose, 4.0 g/liter KH_2_PO_4_, 4.0 g/liter (NH_4_)_2_SO_4_, 0.6 g/liter MgSO_4_·7H_2_O, 0.6 g/liter CaCl_2_, 1.0 g/liter Tween 80, 100 μL trace elements, pH 5.5) in a flask at a final concentration of 1 × 10^6^ spores/mL and cultivated in a shaker with 150 rpm at 28°C for 24 h. The collected hyphae were then inoculated into Avicel medium (1.0 g/liter Avicel, 4.0 g/liter KH_2_PO_4_, 4.0 g/liter (NH_4_)_2_SO_4_, 0.6 g/liter MgSO_4_·7H_2_O, 0.6 g/liter CaCl_2_, 1.0 g/liter Tween 80, 100 μL trace elements, pH 5.5) and cultured for 24 h (17). Fungal mycelium was harvested by filtration and used for total DNA extraction for Hi-C sequencing.

DNA extraction was carried out using the chemical method described by Zhao et al. (17). DNA concentration and purity were determined with a spectrophotometer, by measuring *A*_260_ and *A*_260_/*A*_280_, respectively, and DNA quality was determined by electrophoresis on 0.8% (wt/vol) agarose gels.

### PacBio sequencing and assembly.

Fungal DNA was sequenced on a PacBio Sequel sequencer at Beijing Genomics Institute (BGI, Shenzhen, China). The raw reads were filtered to remove adapters and low-quality data to generate subreads that were used for further assembly with a serial of software Falcon version 0.3.0 (https://github.com/PacificBiosciences/falcon), proovread version 2.12 (https://github.com/BioInf-Wuerzburg/proovread), Celera assembler version 8.3 (http://sourceforge.net/projects/wgs-assembler/files/wgs-assembler/wgs-8.3/), GATK version 1.6-13 (http://www.broadinstitute.org/gatk/), and SSPACE_Basic version 2.0 (http://www.baseclear.com/genomics/bioinformatics/basetools/SSPACE). The contigs obtained were further reordered and integrated into the pseudochromosome assembly using the Juicebox assembly tool (47). BUSCO (48) was employed to evaluate the integrity of the genome assembly and the predicted genes.

### Hi-C library construction and sequencing.

P. oxalicum mycelium was fixed in 1% (vol/vol) formaldehyde for 30 min at room temperature, and the cross-linking reaction was stopped by the addition of glycine solution. The cross-linked DNA of the fixed mycelium was extracted by the chemical method previously described (17) and subsequently cut with the restriction endonuclease MboI (New England BioLabs Inc., Ipswich, MA). The cohesive ends obtained were filled with the biotin marker to generate blunt ends, and then ligated by T4 DNA ligase (New England BioLabs Inc.). Proteinase K (Thermo Fisher, Waltham, MA) was employed to break DNA cross-linking. The purified DNA was sheared randomly into DNA fragments 300 to 500 bp long. Biotin-labeled DNA was purified by pulldown with streptavidin-coupled Dynabeads and then subjected to paired-end sequencing by BGI on the MGISEQ-2000 platform with PE150. Two biological replicates were used for each sample.

### Reassembly of genome assisted by Hi-C technique.

Proximity-guided assembly (PGA) with Hi-C technique of P. oxalicum genome from PacBio sequencing by the 3D-DNA pipeline published previously (49). Briefly, the generated clean reads via Hi-C sequencing were mapped into the raw genome of P. oxalicum by PacBio sequencing and *de novo* assembly. Generally, the contigs/scaffolds localized in same chromosome present more Hi-C interactions than those in distinct chromosomes. Based on that, several groups containing numbers of contigs/scaffolds belonging to individual chromosome were clustered. In the individual chromosome, the contigs/scaffolds in close proximity exhibited improved interactions compared to those that were distant. In the obtained groups above, the contigs/scaffolds were reordered. Subsequently, the orientation of each contig/scaffold was determined according to the exaction positions of Hi-C interactions. Consequently, the assisted assembled genome was evaluated and corrected using software Juicer with Juicebox (50).

### Genome annotation and functional analysis.

Gene prediction in the P. oxalicum genome and their functional annotation were carried out according to the method described by Zhao et al. (17). Numerous software packages were employed, including Augustus (http://bioinf.uni-greifswald.de/augustus/), GeneMark (http://exon.gatech.edu/), and GeneWise (https://www.ebi.ac.uk/Tools/psa/genewise/), as well as databases such as GeneOntology (GO; http://geneontology.org/), KEGG (https://www.genome.jp/kegg/), Cluster of Orthologous Groups of Proteins (COG; http://clovr.org/docs/clusters-of-orthologous-groups-cogs/), NR on NCBI (https://www.ncbi.nlm.nih.gov/), InterPro (http://www.ebi.ac.uk/interpro/), and dbCAN (https://bcb.unl.edu/dbCAN2/). Both rRNA and tRNA genes were predicted using RNAmmer (http://gtrnadb.ucsc.edu/). AntiSMASH (https://fungismash.secondarymetabolites.org/#!/start) was used to annotate secondary metabolism gene clusters. The rRNAs and tRNAs were screened by rRNAmmer (version 1.2) and tRNAscan-SE (version 2.0.9), respectively (22, 23).

### Hi-C data analysis.

The sequenced raw reads were filtered and quality-evaluated using software Trimmomatic (51) and FastQC (52) with the default parameters to generate clean reads. The clean reads obtained were mapped onto the genome of P. oxalicum strain HP7-1 assembled as described above with the bowtie2 (53), and the valid pair reads were generated by the hiclib pipeline (54). The reliability of the Hi-C data from the two replicates was accessed with Genome DISCO (20). TADs/globules were identified by calculating the insulation score values using the software cworld-dekker (29). The intra- and interchromosomal interactions at the resolution of 10-kb bins were selected by Fit-Hi-C software (25), with thresholds of *P* value ≤ 0.01, *q* value ≤ 0.01, and contact count > 2. Motif scan was carried out in the JASPAR database (http://jaspar.genereg.net) using MEME Suite (http://meme-suite.org).

### Aggregate chromosome analysis.

Aggregate chromosome analysis was performed by the method described by Hoencamp et al. (24). In brief, each chromosome arm was rescaled to a uniform length, and subsequently the signals from all inter- and intrachromosomal contacts were aggregated.

### Prediction of TF-binding motifs in globule boundaries.

DNA motifs in the globule boundaries that were bound by TFs were predicted by Find Individual Motif Occurrences (FIMO) in MEME Suite (http://meme-suite.org) with default parameters. The TF-binding profiles of fungi from JASPAR CORE database (http://jaspar.genereg.net/) was used as the reference. Consequently, the homologous alignment was employed to search for the orthologs in P. oxalicum.

### Establishment of 3D genomic model of P. oxalicum.

The 3D model of P. oxalicum genome from Hi-C data were predicted by the pastis software using a 20-kb resolution matrix with the multidimensional scaling (MDS) module (55). In the 3D model of P. oxalicum, the regions of centromeres were annotated based on Centurion developed by Varoquaux et al. (56) based on the obtained Hi-C data. In brief, we statistically analyzed the points of all *trans*-contacts. After removing those localized at the ends of chromosomes, the regions that most frequently interacted were selected as the centromeres.

### Data availability.

The assembled genome sequence and Hi-C data of P. oxalicum strain HP7-1 were deposited at DDBJ/ENA/GenBank under the BioProject accession number PRJNA772803 and genome accession number JRVD00000000. The version described in this paper is version JRVD02000000. Transcriptome data of P. oxalicum on Avicel used in this study was available on SRA with an accession number GSE133258 (GSM3904303-05).
