# Supplementary figures and images for: Three-Dimensional Genome Map of the Filamentous Fungus Penicillium oxalicum
Source: Microbiol Spectr. 2022 May 2;10(3):e02121-21. doi: 10.1128/spectrum.02121-21 (PMC9241887; doi:10.1128/spectrum.02121-21)

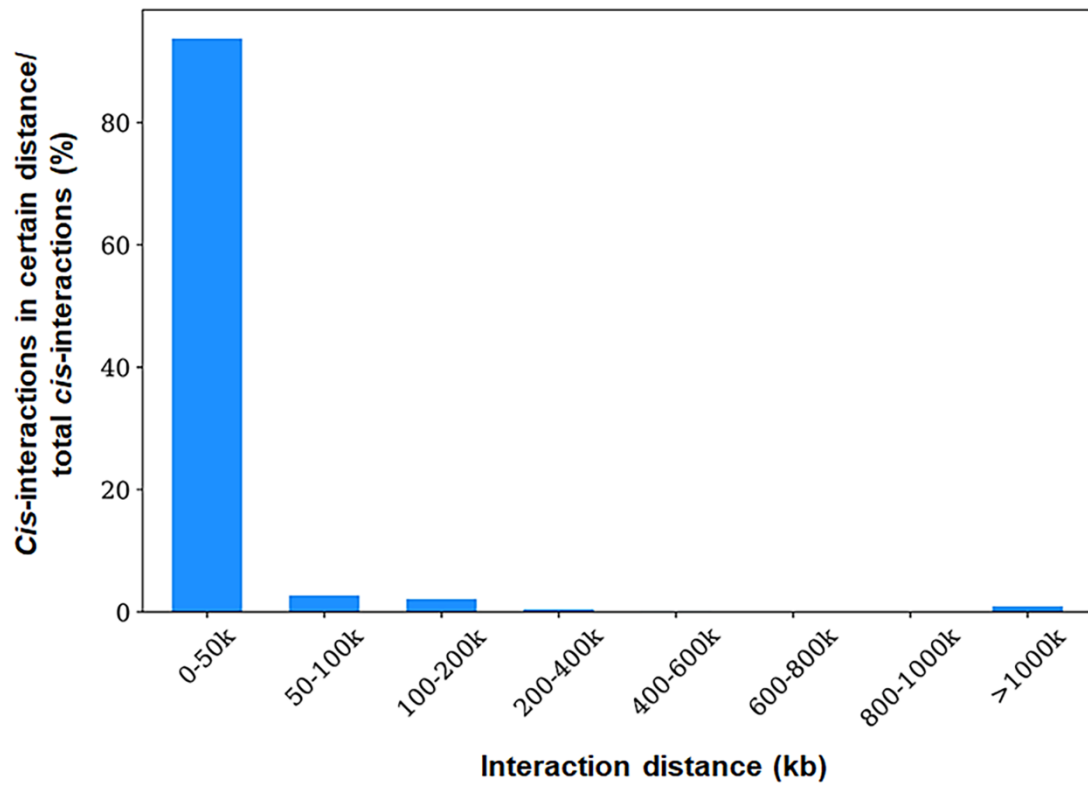

**Fig. S1 Analysis of cis-interactions in certain distances in the genome of *Penicillium oxalicum*.**

Supplement: SUPPLEMENTAL FILE 6 — Supplemental material. Download spectrum.02121-21-s006.pdf, PDF file, 0.2 MB [file spectrum.02121-21-s006.pdf]
